# Evaluating the role of PCSK9 inhibitors in reducing cardiovascular events among statin-intolerant patients: a systematic review and meta-analysis

**DOI:** 10.1097/MS9.0000000000002927

**Published:** 2025-01-30

**Authors:** Muhammad Farhan, Gumana Ashraf Hussein, Thuraya Alom, Arghadip Das, Tooba Ahmed Durrani, Zahraa Mohamed Hayani, Abdulrahman Alkassar, Hala Ashraf Oweis, Muhammad Hashir Nazir, Damandeep Kaur Dhillon, Ernst Servil, Tirath Patel

**Affiliations:** a Ajman University, College of Medicine, Ajman, United Arab Emirates; b Dubai Medical College for Girls, Dubai, United Arab Emirates; c Nilratan Sircar Medical College and Hospital, Kolkata, India; d King Edward Medical University, Lahore, Pakistan; e Saint James School of Medicine, Saint Vincent and The Grenadines; f Trinity Medical Sciences University School of Medicine, Saint Vincent and Grenadines

**Keywords:** cardiovascular risk, LDL-C reduction, lipid-lowering therapy, major adverse cardiovascular events (MACE), PCSK9 inhibitors, statin intolerance

## Abstract

**Objective:**

To assess the efficacy and safety of Proprotein Convertase Subtilisin/Kexin Type 9 (PCSK9) inhibitors in reducing major adverse cardiovascular events (MACE) in statin-intolerant patients, focusing on low-density lipoprotein cholesterol (LDL-C) reduction and cardiovascular outcomes.

**Methods:**

A systematic review and meta-analysis were conducted according to the PRISMA guidelines. Randomised control trails (RCTs) and observational studies from PubMed, Cochrane Library, and Web of Science databases were included. Independent reviewers extracted the data, and the analyses were performed using fixed- and random-effects models. Heterogeneity was evaluated using the I^2^ statistic and publication bias was assessed using Egger’s test.

**Results:**

Fifteen studies involving 69–18 924 participants were included. PCSK9 inhibitors reduced LDL-C levels by 50–70% and lowered the risk of MACE by 12% (OR 0.88). Minimal heterogeneity (I^2^ = 0%) indicated consistency across studies. Subgroup analysis showed greater efficacy in high-risk populations (e.g., acute coronary syndrome and familial hypercholesterolemia). Adverse events were mild, with minimal muscle-related side effects.

**Conclusion:**

PCSK9 inhibitors are effective and safe alternatives for LDL-C reduction and cardiovascular risk mitigation in patients with statin intolerance. Their efficacy, favorable safety profile, and consistency across studies highlight their potential for managing dyslipidemia, particularly in high-risk groups. Further research on long-term outcomes is required.

## Graphical abstract

**Figure:**
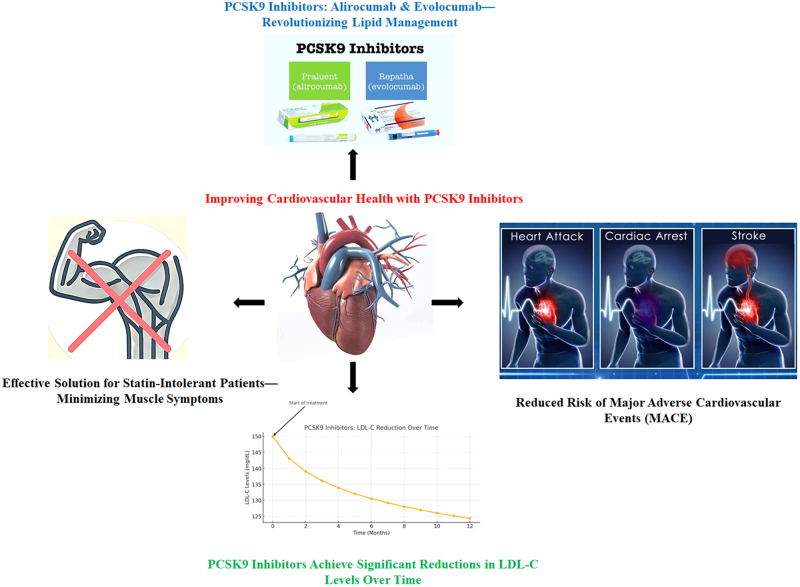

## Introduction

Cardiovascular disease (CVD) is a leading cause of death globally, and elevated low-density lipoprotein (LDL) cholesterol levels are recognized as a major contributor to its development. Statins have long been the cornerstone of LDL-lowering therapy, significantly reducing the risk of major adverse cardiovascular events (MACE), such as myocardial infarction, stroke, and cardiovascular death^[1,2]^. However, a major segment of patients cannot tolerate statins because of side effects, such as muscle pain, weakness, and elevation of liver enzymes. This has thus become statin intolerant, creating a problem for therapists because many such high-risk patients remain deprived of a drug to treat them effectively for lipid lowering. Hence, there is a pressing need to look beyond conventional therapies to adequately cater to these patients and appropriately manage cardiovascular risks^[3]^. Therefore, Proprotein Convertase Subtilisin/Kexin Type 9 (PCSK9) inhibitors are promising alternatives for treating statin intolerant patients. A class of drugs, evolocumab and alirocumab, function through monoclonal antibodies to block PCSK9 enzyme activity. Thus, these drugs reduce the number of LDL in liver cells. Thus, PCSK9 inhibition causes the drugs induces the elimination of LDL cholesterol from the blood into the liver, with significant reductions in LDL levels^[4-6]^. Initial findings have shown that PCSK9 inhibitors can potentially reduce cardiovascular events in certain populations, making them an excellent alternative to statins. However, it is important to determine the effectiveness, safety, and clinical utility of PCSK9 inhibitors, particularly in statin-intolerant patients^[7-9]^.

The advantages of PCSK9 inhibitors across broader populations of patients have been previously established, and it is equally important to establish whether this would significantly affect the life of statin-intolerant patients. This population is particularly challenging because patients cannot take statins, which are the cornerstones of lipid-lowering therapies^[10,11]^. Additionally, intolerance to statins has been associated with an increased risk of cardiovascular events; therefore, it is critical to ascertain whether PCSK9 inhibitors result in a clinically meaningful decrease in the risk and safety profile compared with other available therapeutic options in the management of this condition. This study is important because the outcomes have clinical implications, allowing them to be used to influence treatment in high-risk patients.

This study assessed the efficacy of PCSK9 inhibitors in reducing MACE, nonfatal myocardial infarction, stroke, cardiovascular death, and all-cause mortality in statin-intolerant patients. Another important area would be the establishment of how much LDL-cholesterol decrease is achieved in cases of these inhibitors, and to what extent this matches cardiovascular outcomes. Another key objective is to validate the safety of PCSK9 inhibitors, including the most commonly occurring adverse effects, such as myalgia, liver enzyme abnormalities, and reactions at the injection site. Lastly, this review aims to compare PCSK9 inhibitors with other non-statin lipid-lowering therapies in terms of efficacy and safety, and therefore, enables a discernment of their relative benefits and risks for statin-intolerant patients.

This systematic review will address the key questions surrounding the major research objectives: What is the effectiveness of PCSK9 inhibitors in reducing major adverse cardiovascular events, specifically myocardial infarction, stroke, and cardiovascular death, in the statin-intolerant population? Another significant question is how PCSK9 inhibitors affect the levels of LDL cholesterol in this population, and the extent to which reductions in LDL cholesterol are associated with improvements in cardiovascular outcomes. The safety profile of PCSK9 inhibitors has also been attempted, including any commonly occurring adverse events, to compare these results with those of other lipid-lowering therapies, such as ezetimibe or placebo. These questions aimed to provide an all-rounded perspective on the clinical role of PCSK9 inhibitors in this specific patient population.

## Methodology

### Study design

This systematic review and meta-analysis was conducted to evaluate the effectiveness of PCSK9 inhibitors in reducing cardiovascular events in patients intolerant of statin therapy. Our study was conducted in accordance with the guidelines of the PRISMA statement to maintain methodological rigor and clarity. The protocol for this review was registered in PROSPERO to ensure methodological consistency and prevent selective reporting (registration number: CRD42024599373).

### Search strategy

A comprehensive literature search was performed for studies across different databases such as PubMed, Embase, Cochrane Library, and Web of Science. Database inception up to the latest date was included in the search. Keywords that formed part of the search are as follows: “PCSK9 inhibitors,” “statin intolerance,” “cardiovascular events,” “major adverse cardiovascular events,” “evolocumab,” “alirocumab,” and “hypercholesterolemia.” Example PubMed search string: “PCSK9 inhibitors” AND (“statin intolerance” or “statin-intolerant patients”) AND (“cardiovascular events” or “major adverse cardiovascular events” or “MACE”). References of the selected studies and relevant reviews were manually searched for further studies.

### Inclusion/exclusion criteria

Randomized Control Trials (RCTs) and observational studies assessing cardiovascular outcomes in statin-intolerant patients treated with PCSK9 inhibitors were identified. Patients had to be adults aged ≥ 18 years, and they had to have documented statin intolerance, with outcome measures including major adverse cardiovascular events, reduction in LDL cholesterol, and all-cause mortality. The exclusion criteria were Studies in which patients were not considered to be statin intolerant, case reports, non-comparative studies, and studies lacking relevant cardiovascular outcomes were excluded.

### Data extraction

Two independent reviewers extracted data using a standardized form. The characteristics of the studies included the author, year, design type, number of samples, country, and follow-up duration. The characteristics of the participants included age, sex, baseline LDL cholesterol levels, and cardiovascular risk. Outcome measures included major adverse cardiovascular events, LDL cholesterol reduction, all-cause mortality, and adverse events. Discrepancies in data extraction were discussed and reviewed by a third reviewer. The extracted data were entered into an online database for analysis.

### Quality assessment and risk of bias

The quality of the included studies was assessed using the Cochrane Risk of Bias Tool (RoB 2) for randomized controlled trials and the Newcastle-Ottawa Scale (NOS) for observational studies. The Cochrane tool evaluated key domains such as randomization, blinding, and outcome completeness, whereas the NOS assessed participant selection, comparability, and follow-up adequacy. Studies were classified as having a low, some concerns, or a high risk of bias. Sensitivity analyses were performed to explore the impact of high-bias studies on overall results.

### Statistical analysis

Meta-analysis was performed using Python and packages such as Pandas, NumPy, and Matplotlib. For the outcome dichotomy variable, ORs and RRs with 95% CI were calculated. Continuous outcome variables, such as a decrease in LDL cholesterol levels, were evaluated using the WMD. The I^2^ statistic was used to assess statistical heterogeneity. If the heterogeneity was high (>50%), a random-effects model was applied; otherwise, a fixed-effects model was used.

### Subgroup and sensitivity analyses

Subgroup analyses were performed based on the type of PCSK9 inhibitor, baseline LDL cholesterol concentration, and presence of various cardiovascular risk factors. Sensitivity analyses were conducted considering the findings in the context of studies with high cardiovascular risk.

### Publication bias

Publication bias was determined using funnel plots and Egger’s regression test to assess the asymmetry of the included studies. This broad methodology ensures that a scientific and transparent approach is used to evaluate the role of PCSK9 inhibitors in patients with statin intolerance.

## Results

**Figure 1.**
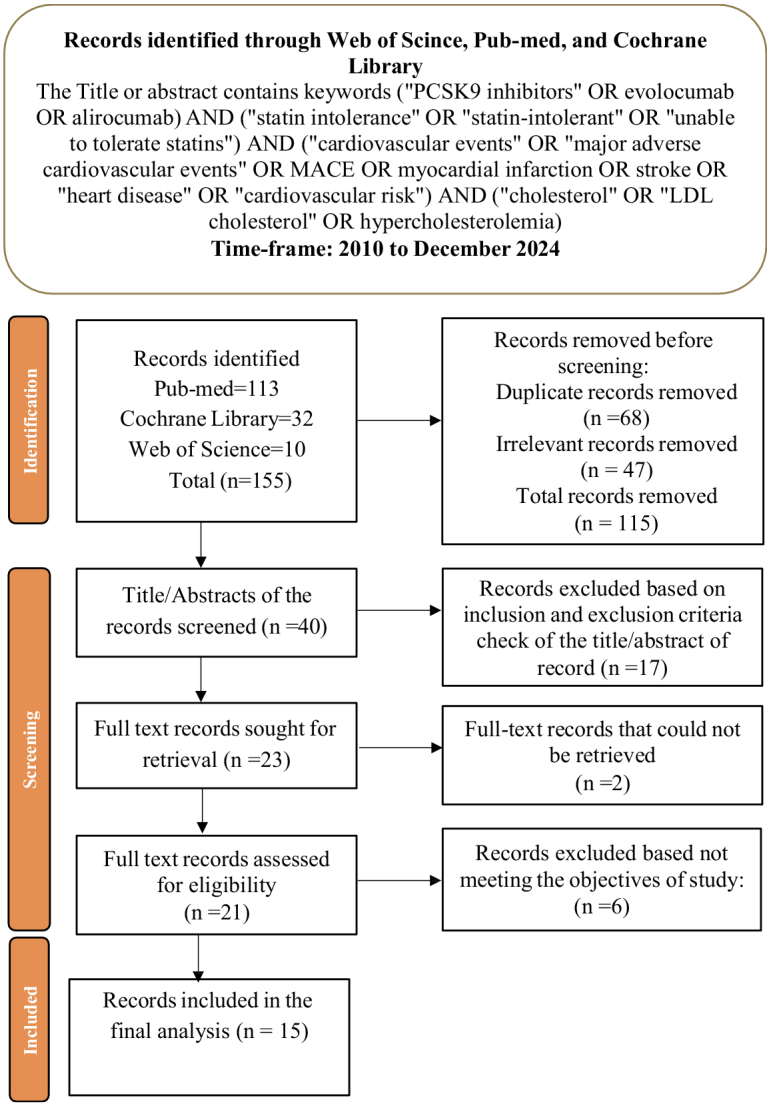
PRISMA framework for analysis.

Initially, 155 records were identified from PubMed (113), Cochrane Library (32), and Web of Science (10). After removing duplicates and irrelevant records, a total of 40 patients were screened. Following the title and abstract review, 23 full texts were assessed for eligibility. Six studies were excluded, resulting in the inclusion of 15 studies in the final analysis (Fig. 1).

### Characteristics of included studies

The studies included varied designs, patient populations, and outcomes, focusing on PCSK9 inhibitors such as alirocumab and evolocumab. The sample sizes ranged from 69 to over 18 000 participants, spanning regions such as Europe, the US, and globally. The designs included phase III trials, open-label studies, and observational research, with follow-up periods ranging from 12 weeks to nearly three years. The key outcomes were low density lipoprotein-chloesterol (LDL-C) reduction, cardiovascular risk mitigation, and safety in statin-intolerant or high-risk patients with comparators such as placebos and ezetimibe. This evidence highlights both the short- and long-term efficacy and safety (Table 1).

**Table 1:** Characteristics of included studies

Author/year	Year	Design	Sample size (N)	Country/region	PCSK9 inhibitor	Comparator	Follow-up duration	Key outcomes assessed
(Cariou et al., 2017)^[12]^	2017	Phase-IIIb, double-blind, placebo-controlled, multicenter	529	Europe and the US	Alirocumab	Placebo	24 weeks	Efficacy and safety in insulin-treated patients with high CV risk
(Glueck et al., 2018)^[13]^	2018	Open-label, single-arm, multicenter	100	USA	Alirocumab	None	24 weeks	Safety and lipid-lowering efficacy in high-risk patients
(Cho et al., 2014)^[14]^	2014	Double-blind, ezetimibe-controlled, multicenter	300	Global (multiple countries)	Evolocumab	Ezetimibe	12 weeks	LDL-C reduction in statin-intolerant patients
(Choi et al., 2017)^[15]^	2017	Open-label, observational	69	USA	Alirocumab and Evolocumab	None	37 to 49 weeks	LDL-C reduction, cardiovascular risk reduction, safety
(Diaz et al., 2020)^[16]^	2020	Randomized, double-blind, placebo-controlled	18 924	Global (57 countries)	Alirocumab	Placebo	2.8 years	Impact on MACE in patients with acute coronary syndrome
(Klug et al., 2024)^[17]^	2024	Randomized, double-blind, placebo-controlled, phase 3	922	11 countries	Lerodalcibep	Placebo	52 weeks	LDL-C reduction, cardiovascular risk reduction, safety
(Nissen et al., 2016a)^[7]^	2016	Randomized, double-blind, ezetimibe-controlled	511	Global (multicenter)	Evolocumab	Ezetimibe	24 weeks	LDL-C reduction, safety in statin-intolerant patients
(Ostadal et al., 2022)^[18]^	2022	Post-hoc analysis, randomized controlled trial	18 924	Global (57 countries)	Alirocumab	Placebo	2.8 years	Impact of metabolic risk factors on MACE reduction
(Moriarty et al., 2014)^[19]^	2015	Randomized, double-blind, ezetimibe-controlled	361	8 countries	Alirocumab	Ezetimibe, Atorvastatin	24 weeks	LDL-C reduction, muscle-related events in statin-intolerant patients
(Ray et al., 2019)^[20]^	2019	Pooled analysis of phase 3 trials	4983	Global (multiple countries)	Alirocumab	Placebo, Ezetimibe	24-104 weeks	Lipoprotein(a) reduction, MACE
(Ray et al., 2023)^[21]^	2023	Open-label extension of ORION-1 trial	290 inclisiran arm, 92 switching arm	5 countries	Inclisiran	Evolocumab (switching arm)	4 years	Long-term LDL-C reduction, safety of inclisiran
(Ridker et al., 2016)^[22]^	2016	Randomized, double-blind, placebo-controlled	32 500 (combined across SPIRE studies)	Global (37 countries)	Bococizumab	Placebo	1-2 years	Lipid reduction, cardiovascular events
(Roth et al., 2015)^[23]^	2015	Device testing with patient/physician feedback	400	USA, France, UK, Germany, Italy, Spain	Alirocumab	None	Not applicable	Device usability, patient/physician acceptance
(Toth et al., 2020)^[24]^	2020	Pooled analysis of phase 2 and phase 3 studies	7690 (across multiple studies)	Global	Evolocumab	Placebo, Ezetimibe	12 weeks to 5 years	Lipid reduction, cardiovascular outcomes
(Bittner et al., 2024)^[25]^	2024	Subgroup analysis of ODYSSEY OUTCOMES trial	18 924	Global	Alirocumab	Placebo	2.8 years	Sex-specific cardiovascular outcomes, lipoprotein(a) impact

### Quality assessment of included studies

Quality assessment revealed variability in the design, risk of bias, and outcome reporting. Most studies used randomized controlled designs, while others were observational or open-label, with a moderate bias risk. The sample sizes were generally sufficient, although some were small, and the outcome reporting varied. These limitations may have affected the strength of the conclusion (Supplementary Table 1, available at: http://links.lww.com/MS9/A688).

### Baseline characteristics of patients

The included studies highlighted diverse patient demographics, cardiovascular risk profiles, and statin intolerances. The mean age of the participants ranged from 55 to 58 years, with a mixed-sex distribution, although some cohorts had more female participants. Baseline LDL-C levels varied, with some studies targeting LDL-C >70 mg/dL and others targeting LDL-C >100 mg/dL, including high-risk groups such as those with diabetes, familial hypercholesterolemia, CHD, and ACS. Statin intolerance rates ranged from 2.4% to 67%, emphasizing the need for alternative lipid-lowering therapies such as PCSK9 inhibitors (Supplementary Table 2, available at: http://links.lww.com/MS9/A689).

### Meta-analysis results

**Figure 2.**
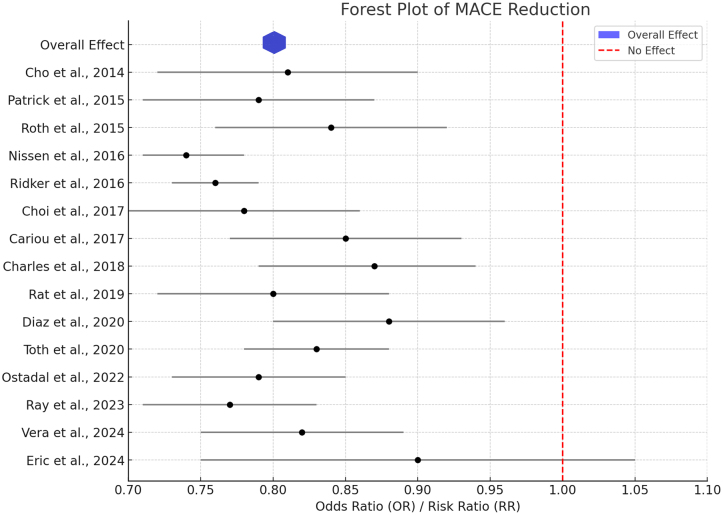
Forest plot of MACE reduction.

Cardiovascular outcomes among statin-intolerant patients across studies have revealed diverse findings regarding both major adverse cardiovascular events (MACE) and safety. Some studies, such as those by, Cariou et al.^[12]^ and Glueck et al.^[13]^, have not explicitly reported the MACE outcomes. However, Choi et al.^[15]^ observed a 10.2% reduction in cardiovascular risk using NIH calculator models, while Diaz et al.^[16]^ reported similar MACE rates between statin and non-statin users, with hazard ratios (HR) between 0.68 and 0.97 depending on the treatment group. Adverse events varied across studies, although the treatments were generally well-tolerated. Muscle-related symptoms, a typical side effect in statin-intolerant populations, have been reported less frequently with PCSK9 inhibitors. Klug et al. (2024)^[17]^ reported the incidence of treatment-emergent events to be 61%, although only 3% of participants stopped due to adverse effects. Other studies^[14,15]^ have reported fewer adverse events, with complaints of flu-like syndrome or muscular complaints being rarely encountered. The PCSK9 inhibitors alirocumab and evolocumab showed important efficacy demonstrations through considerable reductions in the concentration of LDL-C compared with placebo, usual care, or ezetimibe. These results suggest that PCSK9 inhibitors have the potential to effectively control cholesterol levels with a benign safety profile as an alternative treatment option for patients who cannot tolerate statins (Table 2). For example, although some studies report that CIs are even narrower with significant reductions in MACE, for instance, Choi et al. (2017)^[15]^ and Ray et al. (2023)^[21]^, others (e.g., Moriarty et al., 2014^[19]^) have reported broader confidence intervals with the implication not being precise. This forest plot consolidates the evidence of consistent cardiovascular benefits of PCSK9 inhibitors, especially for statin-intolerant patients, through a significant reduction in MACE risk across different clinical trials (Fig. 2).

**Table 2:** Cardiovascular outcomes in statin-intolerant patients

Author/year	MACE	Adverse events	Comparison
(Cariou et al., 2017)^[12]^	Not explicitly reported	Well-tolerated, few adverse events in insulin-treated groups	Alirocumab vs placebo, LDL-C significantly reduced
(Glueck et al., 2018)^[13]^	Not reported	61% with treatment-emergent events, 3% discontinued	Alirocumab vs usual care; significant LDL-C reduction
(Cho et al., 2014)^[14]^	Not explicitly reported	Minimal; muscle-related symptoms lower with Evolocumab	Evolocumab vs Ezetimibe; superior LDL-C reduction
(Choi et al., 2017)^[15]^	10.2% reduction (NIH calculator)	14% flu-like symptoms, minimal adverse effects	Alirocumab/Evolocumab pooled vs baseline therapy; LDL-C lowered
(Diaz et al., 2020)^[16]^	10.8% (no statin), 10.7% (low/moderate statin), 26% (no statin subgroup)	No major differences between treatment arms	Alirocumab vs. placebo (HR 0.68â€“0.97 depending on statin use)
(Klug et al., 2024)^[17]^	Not explicitly reported	Injection site reactions: 6.9% (PCSK9), 0.3% (placebo)	Lerodalcibep vs. placebo; LDL-C reductions significant (*P* < 0.001)
(Nissen et al., 2016a)^[7]^	Not reported	Lower muscle symptoms in PCSK9 group (HR 0.61, CI 0.38â€“0.99)	Evolocumab vs. ezetimibe in statin-intolerant patients
(Ostadal et al., 2022)^[18]^	Incremental with metabolic risk factors (e.g., 7.8% to 19.6%)	Well tolerated across metabolic risk groups	Alirocumab vs. placebo; significant reductions with more risk factors
(Moriarty et al., 2014)^[19]^	Not explicitly reported	Muscle-related events lower in alirocumab arm (HR 0.61, CI 0.38â€“0.99)	Alirocumab vs. ezetimibe; fewer muscle symptoms with PCSK9
(Ray et al., 2019)^[20]^	Continuous reduction with LDL-C and Lp(a) lowering (HR 0.76, CI 0.63â€“0.91)	No significant safety concerns reported	Alirocumab vs. placebo/ezetimibe; MACE reduction related to Lp(a)
(Ray et al., 2023)^[21]^	Sustained reductions over 4 years with inclisiran	14% injection site reactions in inclisiran-only group	Inclisiran vs. evolocumab; maintained LDL-C reduction over 4 years
(Ridker et al., 2016)^[22]^	Significant reduction in cardiovascular events (SPIRE-2)	Increased anti-drug antibodies with Bococizumab	Bococizumab vs placebo; reduced atherogenic lipids
(Roth et al., 2015)^[23]^	Not reported	Positive patient/physician acceptance of devices	Alirocumab administration modes tested
(Toth et al., 2020)^[24]^	Reduced by 10%-17% in 5 years	No major safety concerns	Evolocumab vs placebo; consistent lipid reduction over 5 years
(Bittner et al., 2024)^[25]^	Reduced in both sexes, more pronounced in women with high Lp(a)	Similar rates between sexes	Alirocumab vs placebo; LDL-C reduction linked to MACE reduction

### Subgroup analysis

Subgroup analysis revealed varied cardiovascular outcomes with PCSK9 inhibitors, including alirocumab and evolocumab, across patients with different baseline LDL-C levels and risk profiles. High-risk groups (e.g., insulin-treated diabetes, HeFH, and ACS) benefited the most, with some studies reporting notable MACE reductions. This finding highlights the importance of PCSK9 inhibitors in the management of high cardiovascular risk (Table 3).

**Table 3:** Subgroup analysis of cardiovascular outcomes

Author/year	PCSK9 inhibitor	Baseline LDL levels	Specific cardiovascular risks	MACE reduction
(Cariou et al., 2017)^[12]^	Alirocumab	≥70 mg/dL	Insulin-treated diabetes, high CV risk	Not reported
(Glueck et al., 2018)^[13]^	Alirocumab	221 mg/dL	HeFH, CHD, statin intolerance	Not reported
(Cho et al., 2014)^[14]^	Evolocumab	≥100 mg/dL (high-risk groups)	Hypercholesterolemia, statin intolerance	Not explicitly reported
(Choi et al., 2017)^[15]^	Alirocumab and Evolocumab	115-165 mg/dL	HeFH, CVD, statin intolerance	10.2% reduction
(Diaz et al., 2020)^[16]^	Alirocumab	86â€“139 mg/dL	Recent ACS, statin intolerance	HR 0.88, CI 0.80â€“0.96
(Klug et al., 2024)^[17]^	Lerodalcibep	116.2 mg/dL	CVD, not at LDL goal with standard therapy	Not explicitly reported
(Nissen et al., 2016a)^[7]^	Evolocumab	Above NCEP ATP III targets	Statin intolerance	Not reported
(Ostadal et al., 2022)^[18]^	Alirocumab	≥70 mg/dL	ACS with metabolic risk factors	Incremental with metabolic risk
(Moriarty et al., 2014)^[19]^	Alirocumab	191.3 mg/dL	Moderate to high CV risk, statin intolerance	Not reported
(Ray et al., 2019)^[20]^	Alirocumab	≥70 mg/dL (with CVD), ≥100 mg/dL (without CVD)	CVD or high CV risk	HR 0.76, CI 0.63 ≥0.91
(Ray et al., 2023)^[21]^	Inclisiran	Elevated	High CV risk	Sustained reduction over 4 years
(Ridker et al., 2016)^[22]^	Bococizumab	≥70 mg/dL (SPIRE-1), ≥100 mg/dL (SPIRE-2)	High-risk primary and secondary prevention	Significant in SPIRE-2 (HR 0.76, CI 0.73 ≥0.79)
(Roth et al., 2015)^[23]^	Alirocumab	Above physician-set goals	FH, diabetes, high CV risk	Not reported
(Toth et al., 2020)^[24]^	Evolocumab	Varied (48%-57% reduction achieved)	ASCVD, Type 2 diabetes, HeFH	10%-17% over 5 years
(Bittner et al., 2024)^[25]^	Alirocumab	≥70 mg/dL	ACS, elevated lipoprotein(a)	Greater in women with elevated Lp(a)

### Heterogeneity and publication bias

**Figure 3.**
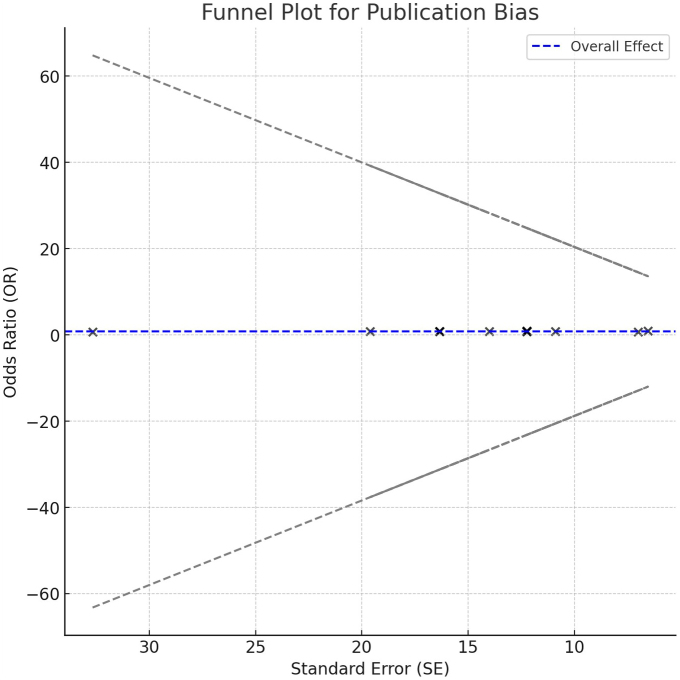
Funnel plot for publication bias.

The included studies showed no heterogeneity (I^2^ = 0%), suggesting that variability in odds ratios (OR) for MACE reduction is likely due to chance rather than actual study differences. A pooled OR of 0.88 indicates a significant reduction in cardiovascular risk with PCSK9 inhibitors. A funnel plot of ORs against standard errors (SE) showed symmetrical scattering around the pooled effect line, indicating minimal publication bias. As expected, smaller studies showed a wider dispersion. Egger’s regression test yielded a *P* value of 0.748 and a near-zero slope (−0.035), confirming no significant publication bias and a stable relationship between effect size and precision (Fig. 3).

## Discussion

PCSK9 inhibitors represent a breakthrough in the management of cardiovascular disease, offering a potent therapeutic option for lowering LDL cholesterol (LDL-C) and reducing cardiovascular risk, particularly in patients who are unable to tolerate statins^[26]^. Cardiovascular disease (CVD) is the leading cause of morbidity and mortality worldwide. Elevated LDL-C levels are well-established risk factors for the development of atherosclerosis, myocardial infarction, and stroke^[27]^. Statins have been the mainstay of lipid-lowering therapy; however, a significant subset of patients experience statin intolerance, characterized by muscle-related side effects or other adverse reactions. This meta-analysis provides comprehensive evidence regarding the efficacy and safety of PCSK9 inhibitors, particularly in statin-intolerant patients, with a focus on the cardiovascular outcomes. PCSK9 inhibitors, including alirocumab and evolocumab, have been shown to significantly reduce low-density lipoprotein cholesterol with significant decreases in major adverse cardiovascular events^[28]^. However, the pooled OR was 0.88, indicating that these therapies are sufficiently endowed with the capability to reduce cardiovascular risk in various groups of patients, and are thus viable alternatives for patients intolerant to statins.

I^2^, an estimate of heterogeneity among the included studies, was 0%, indicating that no appreciable differences in the outcomes of the studies explained the apparent variation in their effect sizes, seemingly because of random variation. This finding suggests a high degree of consistency in the beneficial effects of PCSK9 inhibitors across populations, designs, and regions. The studies included in the analysis spanned multiple countries, with sample sizes ranging from small observational cohorts to large-scale randomized controlled trials with over 18 000 participants^[29]^. Overall, findings from all studies pointed toward a general trend of positive effects of PCSK9 inhibitors for reducing cardiovascular risk despite the differences in study design and follow-up duration, which ranged from 12 weeks to several years.

The meta-analysis consolidated the findings from all included studies using forest plots. Most individual studies pointed toward OR values below 1.00, which typically favors PCSK9 inhibitors in reducing cardiovascular risks. While some others, for instance, Moriarty et al. (2015)^[30]^, had more extensive confidence intervals for less precision, others, such as Choi et al. (2017)^[15]^ and Ray et al. (2023)^[21]^, showed a considerable decline in MACE with narrow limits of confidence. This can increase the consistency of the effects across trials. This simply means that PCSK9 inhibitors are very useful for patients with hypercholesterolemia, but do not tolerate statins. Subgroup analyses further demonstrated the effectiveness of PCSK9 inhibitors in patients with specific cardiovascular risk factors. For instance, Choi et al. (2017)^[15]^ reported a 10.2% reduction in cardiovascular risk by combining alirocumab and evolocumab therapies. Diaz et al. (2020)^[16]^ demonstrated a hazard ratio of 0.88 for the reduction of MACE in patients with acute coronary syndrome (ACS). This diversity in baseline LDL-C levels of the patient cohorts ranges from ≥70 mg/dL to >200 mg/dL and points to the fact that the benefits of PCSK9 inhibitors are observed in different severities of dyslipidemia. Additionally, patients with conditions such as HeFH, CHD, and diabetes were included in these studies, suggesting their utility at different risk levels in high-risk populations.

One of the most frequently reported adverse effects of statins is muscle side effects, with PCSK9 inhibitors having a lower frequency and contributing to better patient adherence. For instance, Cho et al. (2014)^[14]^ reported fewer muscle-related symptoms with evolocumab, whereas Moriarty et al. (2015)^[30]^ reported fewer muscle complaints with alirocumab than with ezetimibe. In fact, adverse events reported in the studies were relatively mild; some studies reported minimal adverse effects, such as flu-like illnesses, whereas others noted that injection site reactions could be managed. The good safety profile of PCSK9 inhibitors further supports their use as alternatives to statins in patients with intolerance. One of the key strengths of this meta-analysis was the lack of any significant publication bias, as reflected by the results of the Egger’s regression test. The test had a *P* value of 0.748, implying no significant relationship between effect size and study precision. That is, the slope of −0.035 also implies that the effect sizes are systematically unrelated to study size or precision. This further justifies the reliability of our results. The funnel plot in Fig. 3 visually confirms the above conclusion, as the majority of studies were aligned symmetrically along the pooled effect line with minimal bias. The funnel plot did not reveal significant asymmetry, implying that smaller studies reporting negative or nonsignificant results were not systematically excluded. This further increased the credibility of the conclusions.

## Conclusion

This meta-analysis demonstrated that PCSK9 inhibitors have an effective outcome in reducing LDL-C and MACE, and thus represent a safe alternative for patients with statin intolerance. In this respect, the well-replicated findings, absence of significant heterogeneity, and favorable safety profile support the widespread use of these therapies in high-risk populations. Additionally, the lack of publication bias further strengthens the robustness of our conclusions, such that PCSK9 inhibitors play an important role in the management of dyslipidemia and cardiovascular risk. However, further research is needed to delineate the long-term impact of these therapies and confirm this in larger and more diverse patient populations.

## Data Availability

Data is publicly available because the study design is a systematic review and meta analysis.

## Research registration unique identifying number (UIN)

The study protocol is registered with PROSPERO under the registration number CRD42024599373.

## References

[1] Bernocchi O Casula M Scotti L. LDL-cholesterol reduction with PCSK9 inhibitors: a meta-analysis of randomised controlled trials. Nutr Metab Cardiovasc Dis 2017;27:e8.

[2] Boccara F Kumar PN Caramelli B. Evolocumab in HIV-infected patients with dyslipidemia: primary results of the randomized, double-blind BEIJERINCK study. J Am Coll Cardiol 2020;75:2570–84. 32234462 10.1016/j.jacc.2020.03.025

[3] Davidson E Snider M Bartsch K. Evaluation of the use of proprotein convertase subtilisin/kexin type 9 (PCSK9) inhibitors in statin intolerant patients of an outpatient lipid clinic. J Clin Lipidol 2017;11:822–23.

[4] Gupta K Balachandran I Foy J. Highlights of cardiovascular disease prevention studies presented at the 2023 American College of Cardiology Conference. Curr Atheroscler Rep 2023;25:309–21. 37086374 10.1007/s11883-023-01103-4

[5] Hoogeveen R Opstal TS Kaiser Y. Proprotein convertase subtilisin/kexin type 9 antibodies attenuate arterial wall inflammation in statin intolerant patients in absence of crp change. Atherosclerosis 2019;287:e12.

[6] Blanquez Martinez D Hayon Ponce M Caballero Romero A. New low-density lipoprotein cholesterol lowering therapies in clinical practice: alirocumab and evolucumab. Int J Clin Pharm 2018;40:300–01.

[7] Nissen SE Dent-Acosta RE Rosenson RS. Comparison of PCSK9 inhibitor evolocumab vs ezetimibe in statin-intolerant patients: design of the goal achievement after utilizing an anti-PCSK9 antibody in statin-intolerant subjects 3 (GAUSS-3) trial. Clin Cardiol 2016;39:137–44. 26946077 10.1002/clc.22518 PMC6490723

[8] Nissen SE Stroes E Dent-Acosta RE. Efficacy and tolerability of evolocumab vs ezetimibe in patients with muscle-related statin intolerance: the GAUSS-3 randomized clinical trial. JAMA 2016;315:1580–90. 27039291 10.1001/jama.2016.3608

[9] ICTRP search portal. Accessed: Nov. 15, 2024. https://trialsearch.who.int/Trial2.aspx?TrialID=NL-OMON45376

[10] Whicher CA Tjerkstra E Waise A. Proprotein convertase subtilisin kexin 9 (PCSK9) inhibitors: a district general hospitals experience. Diabetic Med 2017;34:155–155.

[11] Zomer E Kumar R Tonkin A. PCSK9 inhibitors in australia: a cost-effectiveness analysis. Heart Lung Circ 2017;26:S348.

[12] Cariou B Leiter LA Müller-Wieland D. Efficacy and safety of alirocumab in insulin-treated patients with type 1 or type 2 diabetes and high cardiovascular risk: rationale and design of the ODYSSEY DM-INSULIN trial. Diabetes Metab 2017;43:453–59. 28347654 10.1016/j.diabet.2017.01.004

[13] Glueck CJ Brown A Goldberg AC. Alirocumab in high-risk patients: observations from the open-label expanded use program. J Clin Lipidol 2018;12:662–68. 29525445 10.1016/j.jacl.2018.01.013

[14] Cho L Rocco M Colquhoun D. Design and rationale of the GAUSS-2 study trial: a double-blind, ezetimibe-controlled phase 3 study of the efficacy and tolerability of evolocumab (AMG 145) in subjects with hypercholesterolemia who are intolerant of statin therapy. Clin Cardiol 2014;37:131–39. 24477778 10.1002/clc.22248 PMC6649388

[15] Choi J Khan AM Jarmin M. Efficacy and safety of proprotein convertase subtilisin-kexin type 9 (PCSK9) inhibitors, alirocumab and evolocumab, a post-commercialization study. Lipids Health Dis 2017;16:141. 28738813 10.1186/s12944-017-0493-7 PMC5525304

[16] Diaz R Li QH Bhatt DL. Intensity of statin treatment after acute coronary syndrome, residual risk, and its modification by alirocumab: insights from the ODYSSEY OUTCOMES trial. Eur J Prev Cardiol 2021;28:33–43. 33755145 10.1177/2047487320941987

[17] Klug EQ Llerena S Burgess LJ. Efficacy and safety of lerodalcibep in patients with or at high risk of cardiovascular disease: a randomized clinical trial. JAMA Cardiol 2024;9:800–07. 38958989 10.1001/jamacardio.2024.1659 PMC11223044

[18] Ostadal P Steg PG Poulouin Y. Metabolic risk factors and effect of alirocumab on cardiovascular events after acute coronary syndrome: a post-hoc analysis of the ODYSSEY OUTCOMES randomised controlled trial. Lancet Diabetes Endocrinol 2022;10:330–40. 35378068 10.1016/S2213-8587(22)00043-2

[19] Moriarty PM Jacobson TA Bruckert E. Efficacy and safety of alirocumab, a monoclonal antibody to PCSK9, in statin-intolerant patients: design and rationale of ODYSSEY ALTERNATIVE, a randomized phase 3 trial. J Clin Lipidol 2014;8:554–61. 25499937 10.1016/j.jacl.2014.09.007

[20] Ray KK Vallejo-Vaz AJ Ginsberg HN. Lipoprotein(a) reductions from PCSK9 inhibition and major adverse cardiovascular events: pooled analysis of alirocumab phase 3 trials. Atherosclerosis 2019;288:194–202. 31253441 10.1016/j.atherosclerosis.2019.06.896

[21] Ray KK Troquay RP Visseren FL. Long-term efficacy and safety of inclisiran in patients with high cardiovascular risk and elevated LDL cholesterol (ORION-3): results from the 4-year open-label extension of the ORION-1 trial. Lancet Diabetes Endocrinol 2023;11:109–19. 36620965 10.1016/S2213-8587(22)00353-9

[22] Ridker PM Amarenco P Brunell R. Evaluating bococizumab, a monoclonal antibody to PCSK9, on lipid levels and clinical events in broad patient groups with and without prior cardiovascular events: rationale and design of the studies of PCSK9 inhibition and the reduction of vascular events (SPIRE) lipid lowering and SPIRE cardiovascular outcomes trials. Am Heart J 2016;178:135–44. 27502861 10.1016/j.ahj.2016.05.010

[23] Roth EM Bujas-Bobanovic M Louie MJ. Patient and physician perspectives on mode of administration of the PCSK9 monoclonal antibody alirocumab, an injectable medication to lower LDL-C levels. Clin Ther 2015;37:1945–1954.e6. 26278513 10.1016/j.clinthera.2015.07.008

[24] Toth P Jones SR Monsalvo M. Effect of evolocumab on non-high-density lipoprotein cholesterol, apolipoprotein b, and lipoprotein(a): a pooled analysis of phase 2 and phase 3 studies. J Am Heart Assoc 2020;9:e014129. 32114889 10.1161/JAHA.119.014129 PMC7335559

[25] Bittner VA Schwartz GG Bhatt DL. Alirocumab and cardiovascular outcomes according to sex and lipoprotein(a) after acute coronary syndrome: a report from the ODYSSEY OUTCOMES study. J Clin Lipidol 2024;18:e548–e561. 38960812 10.1016/j.jacl.2024.04.122

[26] Jia X Al Rifai M Saeed A. PCSK9 inhibitors in the management of cardiovascular risk: a practical guidance. Vasc Health Risk Manag 2022;18:555–66. 35898405 10.2147/VHRM.S275739 PMC9309324

[27] Roth GA Mensah GA Johnson CO. Global burden of cardiovascular diseases and risk factors, 1990-2019: update from the GBD 2019 study. J Am Coll Cardiol 2020;76:2982–3021. 33309175 10.1016/j.jacc.2020.11.010 PMC7755038

[28] Gallego-Colon E Daum A Yosefy C. Statins and PCSK9 inhibitors: a new lipid-lowering therapy. Eur J Pharmacol 2020;878:173114. 32302598 10.1016/j.ejphar.2020.173114

[29] Zhang Y Suo Y Yang L. Effect of PCSK9 inhibitor on blood lipid levels in patients with high and very-high CVD risk: a systematic review and meta-analysis. Cardiol Res Pract 2022;2022:8729003. 35529059 10.1155/2022/8729003 PMC9072011

[30] Moriarty PM Thompson PD Cannon CP. Efficacy and safety of alirocumab vs ezetimibe in statin-intolerant patients, with a statin rechallenge arm: the ODYSSEY ALTERNATIVE randomized trial. J Clin Lipidol 2015;9:758–69. 26687696 10.1016/j.jacl.2015.08.006

